# Thyroid Arteriovenous Malformation in Hereditary Hemorrhagic Telangiectasia: Insights on Successful Noninvasive Imaging

**DOI:** 10.1210/jcemcr/luae138

**Published:** 2024-08-12

**Authors:** Hisanori Goto, Iyo Tanimura, Yujiro Nakano, Yumie Takeshita, Toshinari Takamura

**Affiliations:** Department of Endocrinology and Metabolism, Kanazawa University Graduate School of Medical Sciences, Kanazawa, 920-8640, Japan; Department of Radiology, Kanazawa University Hospital, Kanazawa, 920-8641, Japan; Department of Endocrinology and Metabolism, Kanazawa University Graduate School of Medical Sciences, Kanazawa, 920-8640, Japan; Department of Endocrinology and Metabolism, Kanazawa University Graduate School of Medical Sciences, Kanazawa, 920-8640, Japan; Department of Endocrinology and Metabolism, Kanazawa University Graduate School of Medical Sciences, Kanazawa, 920-8640, Japan

**Keywords:** thyroid AVM, HHT, 3D-CTA

## Abstract

Hereditary hemorrhagic telangiectasia (HHT) causes arteriovenous malformations (AVMs) in several organs. This report is the first to document and image a thyroid AVM complication in HHT.

A 72-year-old woman with HHT was referred for thyroid nodule evaluation. Ultrasonography showed a hypervascularized nodule in the right thyroid lobe which was initially suspected to be malignant. However, 3-dimensional computed tomography angiography demonstrated a thyroid AVM with abnormal anastomosis of the superior thyroid artery and the inferior thyroid vein.

In the formation of thyroid AVM, here, chronic thyroiditis and hypothyroidism complications may have been a second hit, due to the predisposing first-hit germline mutation.

This report sheds light on overlooked thyroid lesions in HHT and advocates a noninvasive imaging approach in diagnosing thyroid AVMs. Furthermore, this case suggests a potential mechanism of AVM formation in human HHT, possibly supporting the second-hit hypothesis.

## Introduction

Hereditary hemorrhagic telangiectasia (HHT) is an inherited vascular disorder characterized by multiple abnormal connections between artery and vein; it occurs in 1 in 5000 individuals (1). HHT forms small arteriovenous malformations (AVMs) primarily in the skin and mucosal area and large visceral AVMs in the lungs, brain, and liver. However, thyroid AVMs in HHT have not been reported to date.

Generally, the differential diagnosis of hypervascularized thyroid tumors includes—in addition to malignancy—autoimmune thyroiditis, intrathyroidal paraganglioma (2), benign hemangioma (3), capillary hemangioma (4), epithelioid hemangioendothelioma (5), cavernous hemangioma (6), and AVM. Besides malignancy, the other vascular lesions are rare conditions unfamiliar to endocrinologists. Among them, hypervascular AVMs are usually initially suspected as malignancies and subject to unnecessary needle biopsy, which carries the risk of bleeding (7, 8). Furthermore, useful noninvasive imaging methods for thyroid AVMs are lacking due to their rarity.

HHT is caused by loss-of-function mutations in the BMP/ENG/ALK1/SMAD4 signaling pathway, as evidenced by studies involving mice deficient in these genes (9]. However, to manifest, AVM requires second-hit local factors and deletions of these genes. Moreover, in human HHT cases, the second-hit factors leading to AVM formation and enlargement remain unclarified. The patient case report presented here sheds light on these knowledge gaps.

## Case Presentation

A 72-year-old woman with HHT was referred for evaluation of a thyroid nodule. The patient had a history of recurrent epistaxis, oral mucosal telangiectasia, and pulmonary AVM. She also had a family history of HHT; her sister and son had HHT. Thus, together with the clinical findings, she was diagnosed to have definite HHT (10). She had previously undergone coil embolization for a pulmonary AVM. Further, 8 years before this referral, she was diagnosed with primary hypothyroidism with a thyroid-stimulating hormone (TSH) level of 15.60 μIU/mL (15.60 mIU/L; reference range: 0.27-4.20 µIU/mL; 0.27-4.20 mIU/L) and free thyroxine (T4) 0.88 ng/dL (11.35 pmol/L; reference range: 1.00-1.80 ng/dL; 12.87-23.17 pmol/L), and she had been on levothyroxine since then. Serum thyroglobulin (Tg) antibody 505.0 IU/mL and thyroid peroxidase (TPO) antibody ≥ 600.0 IU/mL were consistent with chronic thyroiditis. The patient was referred to our department for a detailed examination of a nodule incidentally noted in the right lobe of the thyroid gland on a computed tomography (CT) scan conducted for follow-up of a pulmonary AVM. The patient did not exhibit any symptoms related to the thyroid lesion.

## Diagnostic Assessment

**Figure 1.**
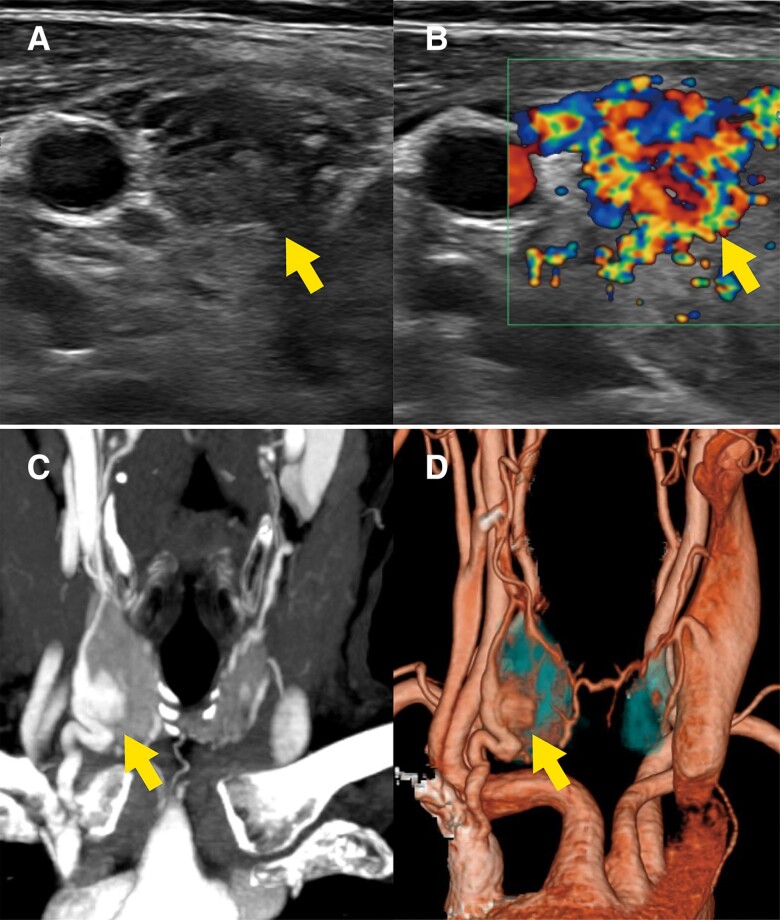
Images of thyroid AVM in the patient reported here. (A) and (B) Ultrasonography revealed a highly vascular, well-circumscribed mass, measuring 11.9 mm × 8.0 mm × 12.3 mm, located in the middle pole of the right thyroid lobe; (C) and (D) The 3-dimensional CT angiographic (3D-CTA) reconstruction clearly illustrated a thyroid AVM, highlighting an abnormal anastomosis between the superior thyroid artery and the inferior thyroid vein. Arrows point to the AVM.

Ultrasonography showed a 12.3 mm hypervascularized nodule in the right lobe of the thyroid gland, which was initially suspected to be malignant (Fig. 1A and 1B). However, a close examination of the ultrasound image suggested that the nodule was contiguous with large blood vessels feeding the thyroid gland, raising the possibility that it was a vascular lesion. Three-dimensional computed tomography angiography (3D-CTA) demonstrated a thyroid AVM with abnormal anastomosis of the superior thyroid artery and the inferior thyroid vein (Fig. 1C and 1D).

Fine needle aspiration (FNA) biopsy was considered when the nodule was suspected to be malignant. However, upon discovering it to be an AVM, FNA was deemed too risky due to the potential for significant bleeding.

We performed a review of the literature and were unable to identify any previously published cases of thyroid AVM associated with HHT (Table 1). A literature survey also revealed that digital subtraction angiography (DSA) successfully visualized 2 prior cases. Thyroid AVMs are challenging to diagnose with thyroid ultrasonography or conventional contrast-enhanced CT. The case presented here is unique in demonstrating the efficacy of 3D-CTA for diagnosing thyroid AVMs (Table 1). Further, the literature review revealed that FNA failed to confirm the AVM diagnosis in 3 cases of thyroid AVMs it was used (Table 1). Moreover, only 2 of the reported thyroid AVM cases presented with concurrent hypothyroidism. We could not find any published cases of AVM complicated by chronic thyroiditis with hypothyroidism in our literature review (Table 1).

**Table 1:** Review of the literature on previously reported thyroid AVMs

Reference	Country	Gender	Age (years)	Diagnostic imaging of thyroid AVMs	Diagnosis by FNA	Thyroid function	Other disorders
(7)	USA	Woman	31	Digital subtraction angiography identified thyroid AVM	Colloid nodule or goiter	Euthyroid	Wyburn-Mason syndrome
(8)	UK	Woman	36	Ultrasonography failed to diagnose AVM preoperatively	Follicular-patterned lesion	Euthyroid	Not reported
(8)	UK	Woman	57	Ultrasonography failed to diagnose AVM preoperatively	Follicular-patterned lesion	Euthyroid	Not reported
(11)	Czech	Man	64	Digital subtraction angiography identified thyroid AVM	Not performed	Euthyroid	Not reported
(12)	USA	Woman	49	Contrast-enhanced CT and ultrasonography failed to diagnose AVM	Not performed	Euthyroid	Endometrial carcinoma
(13)	Germany	Woman	42	Contrast-enhanced MRI identified thyroid AVM	Not performed	Hypothyroidism	Not reported
(14)	Saudi Arabia	Man	32	Digital subtraction angiography identified thyroid AVM	Not performed	Not reported	Not reported
This case	Japan	Woman	72	Three-dimensional CT angiography identified thyroid AVM	Not performed	Hypothyroidism	HHT, Chronic thyroiditis

Abbreviations: AVM, arteriovenous malformations; CT, computed tomography; FNA, fine needle aspiration; HHT, hereditary hemorrhagic telangiectasia; MRI, magnetic resonance imaging.

## Treatment

The patient continued to be treated with levothyroxine.

## Outcome and Follow-Up

This thyroid AVM has been monitored by ultrasonography for 3 years, with no increase in size, compression of surrounding tissues, or signs of cardiac failure. The observation was chosen due to the lack of consensus on optimal treatment, the patient's asymptomatic status, and the AVM's classification as Stage 1 (quiescent stage) according to the Schobinger classification. Stage 1 AVMs may remain stable over time (15). Additionally, embolization alone has a high recurrence rate and combined surgical approaches are invasive with significant risks (16). The patient preferred to avoid invasive treatments. Surgical intervention will be aggressively reconsidered if the AVM enlarges or becomes symptomatic.

## Discussion

Thyroid AVMs are extremely rare, with only 7 reported cases, including the current one (7, 8, 11-14) (Table 1). Further, this report is the first documented case of thyroid AVM complicated by HHT, providing valuable insight into the pathogenesis of AVMs in human HHT. The distinctiveness of this case lies in the occurrence of chronic thyroiditis and associated primary hypothyroidism, which could be implicated in the development of thyroid AVM in HHT. It is hypothesized that HHT is caused by haplo-insufficiency of the mutant gene product because of the causative loss-of-function mutation and the autosomal dominant nature of the disease (17). However, this hypothesis is limited by the fact that despite germline mutations present in all endothelial cells, vascular lesions in HHT are confined to specific vessels. Furthermore, the observed phenotypes are diverse even among patients with the same mutation. These discrepancies support the “second-hit” hypothesis for AVM formation in HHT. The second-hit hypothesis posits that germline mutations constitute the first hit; they induce vascular anomalies in the endothelium. These anomalies predispose the tissue to the development of AVMs when subjected to additional local factors, termed the second hit (9, 18). Although mice deficient in the genes responsible for HHT—*Eng* and *Alk1*—serve as animal models of HHT, local vascular endothelial growth factor (VEGF) overexpression (19, 20) and angiogenic and inflammatory stimuli such as LPS administration (17) are required to induce AVM development in these mice. Probably, the absence of ENG/ALK signaling increases endothelial sensitivity to VEGF, which, in combination with a VEGF-rich local environment, promotes aberrant endothelial proliferation, resulting in AVM formation.

In addition, lymphocytic infiltration surrounding blood vessels is observed in AVMs (21); and a genetic polymorphism encoding TNFα-converting enzyme (ADAM17) is associated with pulmonary AVMs in patients with HHT (22). These indicate that immune response is potentially involved in AVM formation. Inflammation may cause AVM formation as follows: Endoglin is the most commonly mutated protein in HHT. Its expression is reduced by half due to the HHT mutations. Inflammatory cytokines enhance the cleavage of the extracellular domain of endoglin. This exacerbates the reduction in endoglin expression level, dropping it below the threshold necessary to maintain normal vascular structure (23).

Here, the patient presented with chronic thyroiditis, which implies that the thyroid gland was in a state of chronic inflammatory state. In addition, the patient presented with primary hypothyroidism, evidenced by previously elevated TSH levels. TSH stimulates the synthesis of VEGF from follicular cells of the thyroid gland (24). Therefore, in the patient presented here, it is plausible that chronic inflammation of the thyroid gland and local VEGF induction by TSH led to progressive development of the thyroid AVM.

Because thyroid AVMs are extremely rare and may have been overlooked in HHT, the thyroid gland has not been reported as an organ affected by HHT. Considering the relatively high prevalence of chronic thyroiditis—which may contribute to the “second hit” in the formation of thyroid AVMs—it is plausible that more thyroid AVMs remain undetected in patients with HHT.

The case presented here, including the literature review, provides important insights into thyroid AVM diagnosis. As the appropriate diagnostic procedures for AVM and malignancies are quite different, we suggest that while diagnosing hypervascularized thyroid lesions, it is crucial to consider AVMs alongside malignancy in the differential diagnosis. Although FNA is the preferred method when malignancy is the primary concern, it is important to note that FNA does not contribute to the diagnosis of thyroid AVMs (Table 1). Moreover, importantly, this report highlights the usefulness of 3D-CTA in the noninvasive diagnosis of thyroid AVMs. Traditionally, digital subtraction angiography (DSA) is the gold standard for AVM diagnosis, with strong success in identifying thyroid AVMs (Table 1). However, DSA is invasive, technically demanding, and carries risks such as embolism and vascular injury. Alternatively, 3D-CTA is noninvasive, with proven utility in diagnosing brain AVMs, and it helps in preoperative planning by identifying key anatomical features (25). Also, as illustrated here, 3D-CTA effectively visualizes the thyroid AVM structure, including the feeder artery and drainage vein. Thus, it could be a noninvasive diagnostic substitute for DSA.

In summary, this report highlights previously under-recognized thyroid lesions in HHT and recommends appropriate diagnostic procedures for thyroid AVMs. Additionally, it may contribute to the understanding of human HHT pathogenesis by suggesting a potential role for the “second-hit” hypothesis in human HHT.

## Learning Points

- The thyroid gland is also an organ that can be affected by hereditary hemorrhagic telangiectasia (HHT), and some patients with HHT may have undiagnosed thyroid arteriovenous malformations (AVMs).
- Thyroid vascular lesions should be included in the differential diagnosis when encountering a hypervascular thyroid tumor.
- Unlike thyroid malignancies, fine needle aspiration is not a suitable diagnostic tool for thyroid AVMs.
- Three-dimensional computed tomographic angiography (3D-CTA) is an effective noninvasive method for diagnosing thyroid AVMs.
- Management of thyroid AVMs may need to be tailored based on patient symptoms, AVM stage, and the risks associated with treatment options. Surgical procedures combined with embolization should be considered for symptomatic or advanced AVMs.

## Contributors

All authors made individual contributions to authorship. H.G. was involved in the diagnosis and management of the patient. I.T. was responsible for preparing the 3D-CTA images. H.G., I.T., Y.N., Y.T., and T.T. were involved in writing and editing the manuscript. All authors reviewed and approved the final draft.

## Data Availability

Data sharing does not apply to this paper, as no data sets were created in this study.

## Abbreviations

3D-CTA: 3-dimensional computed tomography angiography
AVM: arteriovenous malformation
CT: computed tomography
FNA: fine needle aspiration
HHT: hereditary hemorrhagic telangiectasia
T4: thyroxine
TSH: thyrotropin (thyroid-stimulating hormone)
VEGF: vascular endothelial growth factor
